# Children’s Reality Status Judgments of Digital Media: Implications for a COVID-19 World and Beyond

**DOI:** 10.3389/fpsyg.2020.570068

**Published:** 2020-11-05

**Authors:** Brenna Hassinger-Das, Rebecca A. Dore, Katherine Aloisi, Maruf Hossain, Madeleine Pearce, Mark Paterra

**Affiliations:** ^1^Psychology Department, Pace University, New York, NY, United States; ^2^Crane Center for Early Childhood Research and Policy, The Ohio State University, Columbus, OH, United States

**Keywords:** YouTube, television, mobile phone, reality status, digital media

## Abstract

Even prior to the COVID-19 crisis, one of the children’s most common screen activities was using the video-sharing platform YouTube, with many children preferring YouTube over television. The pandemic has significantly increased the amount of time many children spend on YouTube—watching videos for both entertainment and education. However, it is unclear how children conceptualize the people they see on YouTube. Prior to the pandemic, children 3–8 years old (*N* = 117) were recruited to participate. Children were told that they would see pictures taken from videos and answer questions about them. Children saw three physical photos with the same image of a man and a bird and were told that the photo was (a) from a video on the experimenter’s phone, (b) from a video on television, or (c) from a video on YouTube. They were asked whether the person in the photo was real or not real, which video would be best for learning, and which video they would prefer to watch. Findings indicated that children were marginally less likely to believe that people on YouTube are real than people in a video on a phone, with no difference between beliefs about people on YouTube and television. Notably, these beliefs were similar across the age range tested here. Across all ages, children preferred to watch YouTube more than phone videos and believed that YouTube possessed greater educational value than both phone and television videos.

## Introduction

By the end of March 2020, school closures during the coronavirus disease 2019 (COVID-19) pandemic affected almost 90% of the world’s student learners (Mokhtar and Gross, 2020). As a result, online learning modalities have become a commonplace, even for the youngest of children. This rise in the use of virtual tutoring, educational apps, videoconference classrooms, and YouTube lessons has radically shifted the educational landscape, and these changes may not yield to business-as-usual any time soon—if ever.

Yet, even before COVID-19, children were increasingly using a variety of screen devices on a regular basis. Prepandemic, children’s time using mobile devices had tripled from 2013 to 2017 (Common Sense Media, 2017)—with children under eight using screens for almost 3 h per day. This incredible, widespread international adoption of devices in the homes of children and families has been complemented, at the same time, by similar growth in child-directed content, such as apps and streaming video.

### Preference for YouTube

One of the children’s most common screen activities is using the video-sharing platform YouTube on mobile devices and smart televisions, with many children preferring YouTube over television (Ofcom, 2016). During the COVID-19 pandemic, caregivers noted that YouTube was children’s most commonly used video platform, with over 78% of children watching (ParentsTogether Foundation, 2020). This aligns with research from 2018 where Smith et al. (2018) from the Pew Research Center reported that 81% of United States parents allowed their children under age 11 to watch videos on YouTube. For younger children, 4- to 8-year olds spend approximately 17% of their screen time per day on online video platforms such as YouTube (Common Sense Media, 2017).

Much more so than television, there is an incredible variety of content available on YouTube. For example, YouTube content includes episodes of regular television shows, clips of children and adults playing video games, and music videos. But because the platform allows for user-generated content, children can also watch videos of their friends making slime or baking a cake and now perhaps videos of their teachers reading a storybook or teaching a math lesson. Among children who watch videos online, learning videos emerge as the most-watched category, with 64% of parents reporting that their children watch them often or watch them sometimes (Common Sense Media, 2017). This percentage has likely increased during the pandemic as many teachers and early childhood care providers, as well as authors and celebrities, are now providing YouTube storybook readings and educational videos to support out-of-school instruction (Li and Lalani, 2020). While YouTube has emerged as a popular learning tool for young children, it remains unclear how children conceptualize what they see on YouTube, given that it exists on a platform that contains such diverse content. The importance of investigating this phenomenon has only increased due to the increase in the use of YouTube during the pandemic (Lukovitz, 2020).

### Television Reality Status Judgments

The majority of previous research in this area has focused on adults’ understanding of the reality of television content, while less is known about children’s judgments (e.g., Hall, 2003; Busselle and Bilandzic, 2008). Some studies suggest that 5-year olds take a somewhat all-or-nothing view of television—believing that everyone on television is not real (Wright et al., 1994)—whereas 7-year olds are somewhat better at distinguishing between different types of programs (e.g., news vs. a cartoon). Research also shows a developmental pattern for children’s judgments about the reality of television—where 3- to 4-year olds are more likely than older children to view television pictures as real objects (Flavell et al., 1990) and to confuse characters and the actors portraying them (Goldstein and Bloom, 2015). Work by Li et al. (2015) also found that 4-year olds often underestimate the reality status of real events in videos. Even though they were able to tell that fantastical events were not real, these children also often claimed that real events could not actually happen.

More recent research has shown that children 5–7 years of age are likely to make reality status judgments of television clips with equal accuracy compared with adults, yet behavioral and neuropsychological data demonstrate significant discrepancies (Li et al., 2019). Children took longer periods of time before making a decision about reality status, and output from functional near-infrared spectroscopy (fNIRS) revealed greater activation of the prefrontal cortex for children. Therefore, the authors argued that reality status judgments require increased cognitive resources for children as compared with adults. As in their earlier work (Li et al., 2015), Li et al. (2019) argued that children use their personal experiences with real-life events to make reality judgments, as evidenced by increased activity in the part of the brain associated with working memory and retrieval of memories.

### Digital Media as a Source of Information

Children’s media literacy—or their ability to employ critical thinking to develop individual judgments about the value of media content (Silverblatt and Eliceiri, 1997)—affects how they view digital media as a source of information. Media literacy often increases with age as children gain more experience with various forms of media (Huston and Wright, 1983). Children may also receive school- or home-based instruction regarding how to evaluate media messages.

Researchers have argued that children’s judgments shed light on how they learn from television and other digital media (Bonus and Mares, 2019). Studies show that children are less likely to learn from television when they judge that a show’s content is not real (Mares and Sivakumar, 2014)—suggesting that understanding how children view the reality status of other digital media may have implications for their educational potential. Yet, it is unclear whether these findings also apply to YouTube as a source of information. In many ways, children’s evaluations of information and reality status of media from television and online video platforms, such as YouTube, may be similar. Indeed, no differences were found when exploring preschool children’s responses to video advertising on television versus YouTube (Vanwesenbeeck et al., 2020). Relatedly, children were observed to learn and interact with television and YouTube videos in similar ways, including actively applying information they learned to real-world contexts and sharing learned information with others (Dugan et al., 2010). However, given YouTube’s unique properties of containing both mass-produced and user-generated content, there may also be important differences in how children process and conceptualize content on this popular platform.

### The Present Study

Children like watching YouTube, and the platform’s popularity has grown greatly since its introduction in 2005. Yet, unlike television, little research has examined children’s reality status beliefs about YouTube content. In one qualitative study, Martínez and Olsson (2019) found that 9- to 12-year-old children moved between identifying a YouTuber as a paid celebrity influencer (less real) versus as a young girl (more real), but there is no research to our knowledge that has examined perceptions of the reality status of people on YouTube in younger children. Additionally, little is known about how children view YouTube as a source of information and as an educational resource.

As a result, the current study asks how different media formats (YouTube video, television, and video on a phone) affect 3- to 8-year-olds’ reality status judgments, preferences for videos, and beliefs about the educational value of videos. We hypothesized that children would be more likely to believe that a person in a video from the experimenter’s phone was real compared with a person in a video from television and that these judgments would be more distinct for older children. Given the limited evidence, we did not have a specific hypothesis regarding YouTube; rather, we asked the research question: How do children view the reality status of people in a YouTube video? We also explored children’s preferences for videos from these sources and beliefs about the educational value of the videos but did not have specific hypotheses for these outcomes.

## Materials and Methods

The study design and hypotheses were preregistered on the Open Science Framework and may be accessed at the following link: https://osf.io/wrsbz. The study was powered to detect medium-sized effects (*d* = 0.5).

### Participants

Participants (*N* = 117 children, 53.8% female, 61.5% white; *N* = 101 caregivers; 93.1% mothers, 34.2% college graduates) were recruited at two children’s museums in the United States, one in the Northeast and one in the Midwest (see Table 1 for more demographic information). All children between the ages of 3 and 8 years who were able to see, hear, and understand the stimuli in English were eligible to participate (10.2% of the sample also spoke additional languages—5.1% Spanish; 5.1% other languages). Caregivers were asked to complete a questionnaire about children’s exposure to digital media, types of apps they use, videos/shows they watch, platforms they use to watch, and other related questions. Results indicated that for the children whose caregivers completed the question (*N* = 93), children in the sample watch between 0 and 240 min of television/YouTube per day, with 32.1% of that time dedicated to YouTube. Caregivers reported that out of the total time that their child watches television, they watch television with their child 74.7% of the time on average, while they only watch YouTube with their children 47.0% of the time that their child watches YouTube overall.

**TABLE 1:** Demographics of the sample.

	**% of total sample**
**Age groups**	
3–4-year olds	17.1
5–6-year olds	51.3
7–8-year olds	31.6
**Gender**	
% male	46.2
% female	53.8
**Caregiver**	
Mother	94
Father	6
Other relative	1
**Mother’s education**	
% high school	10.3
% some college	14.5
% college graduate	34.2
% graduate degree	27.4
% no answer	13.7
**Child’s ethnic background**	
% Asian/Pacific Islander	2.6
% African American	8.5
% Latinx	6
% White	61.5
% Other	1.7
% Multiracial	1.7
% No answer	3.4
**Children’s language exposure**	
% English	100
% English and Spanish	5.1
% English and other language (not Spanish or Mandarin/Cantonese)	5.1
	

### Procedure

#### Reality Status Judgments

**FIGURE 1:**
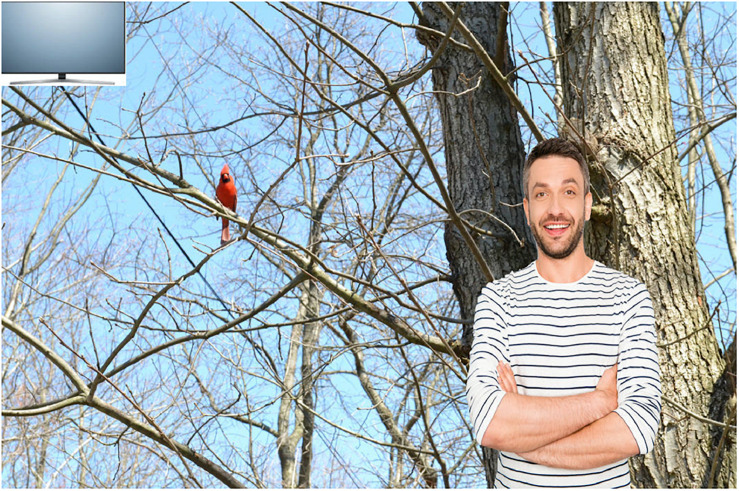
Stimuli image used in the study (with television corner icon). Image of trees and bird: Photographer Robin Moore/used under license from Shutterstock.com. Image of person: Photographer Roman Samborskyi/used under license from Shutterstock.com.

To assess children’s reality status judgments, the research team created an 8 × 10 physical photo with an image of a person that children would likely identify as male along with a nature background featuring a sky, a tree, and a bird (see Figure 1). This composition was chosen because 46% of children 0–8 years of age often/sometimes watch YouTube videos about animals (Common Sense Media, 2017), and popular children’s television shows, such as *Sesame Street* and *Wild Kratts*, feature male animals and male adult characters. Birds are also a type of animal that all children in the sample would have seen in real life, given that birds are common in urban, suburban, and rural areas. Additionally, the image also looked like it could have been taken from a video from someone’s phone. The same image was altered to include an icon in the upper left corner representing the media type (YouTube logo, image of a flat screen television, or an image of a smartphone; see Figure 1 for the image with the television icon). The order in which the experimenter presented the three physical photos representing the three mediums (YouTube, television, phone) was counterbalanced.

xylThe experimenter explained to the children that they were going to look at some pictures taken from videos and answer some questions related to those videos (see Supplementary Materials for the full study protocol). First, the experimenter laid out the three physical photos one at a time, stating with each photo where it came from—YouTube, television, or experimenter’s phone—while referencing the icon in the upper left corner denoting the photo’s source. Then, the experimenter pointed to the first photo (the order of which was counterbalanced across participants) and asked children whether they thought the person in the picture was real or not real. Then, children were asked how confident they were about this judgment (not sure at all, a little bit sure, very sure). Real/not real judgments and confidence ratings were used to create a belief score for each media format from −3 (very sure that it is not real) to +3 (very sure that it is real). Finally, children were asked an open-ended justification question about why they thought that the person was real or not real.

#### Beliefs About Educational Value

Next, children were asked questions about their desire to learn from the videos. First, the experimenter told the children that all of the videos are about birds and reminded them which platform each physical photo was from. Then, the experimenter asked the children which video they thought would be the best for learning about birds. Then, their first choice was removed and they were asked, of the remaining two, which they thought would be best for learning about birds. Then children were asked a justification question about why they thought their first choice would be best for learning about birds and why their last choice would not be as good to learn from. A score was created for each media format, denoting whether that format was selected as a child’s first (1), second (0), or third (−1) choice.

#### Preference

The same sequence as beliefs about educational value was used to ask children about their preference— which video the child would want to watch the most and why.

#### Justifications Coding

The authors generated a coding scheme for the children’s responses to the three justification questions: reality status, educational value, and preference (see Table 2). Then, the lead author trained a research assistant to complete all of the coding. Thirty-two percent of the data were double coded for reliability, and any discrepancies between the lead author and the research assistant were discussed and resolved.

**TABLE 2:** Coding scheme for open-ended responses.

**Category**	**Definition**	**Examples**
Physical-person	The comment is about the physical nature of the person.	“His shirt is green.”; “He looks funny.”
Physical-non-person	The comment is about something else in the picture or about the physical picture itself.	“The bird on the tree is small.”; “The picture looks blurry.”
Medium-subjective	An opinion on the medium (YouTube, television, and phone).	“I think YouTube is the best because I like it the best.”
Medium objective	A fact/description of the medium.	“YouTube is good to learn from because real people put videos on there.”
Device	A comment made about the actual device as opposed to the medium.	“I don’t use the phone because the screen is too small.”
Definitional	The comment is directly addressing the definition of the question (different for each question) or restating the medium.	“I think the guy is real because he looks real.”; “Because it’s on television.”
Personal reference	The comment references something from their personal life.	“I have seen this guy before”; “He looks like my uncle.”
Feelings about photo	Any subjective comments about how the photo makes them feel.	“The photo makes me happy.”
Comment on the photo itself	Any comment referencing the photo itself without adding additional information.	“Because it’s a picture.”
Reality status (only coded for preference question)	Comment about the reality status of that medium.	“I like to watch YouTube because it is real.”; “I do not like to watch television because it is not real.”
Educational information (only coded for preference question)	Comment about the educational quality of the medium.	“I like to watch YouTube because I can learn the most from it.”
I don’t know	Child stated that they did not know why they chose their answer.	“I don’t know.”
Does not fit/miscellaneous	The comment does not fit into any of the previous categories.	
		

## Results

### Reality Status Judgments

#### How Does Media Format Affect Children’s Reality Status Judgments?

**FIGURE 2:**
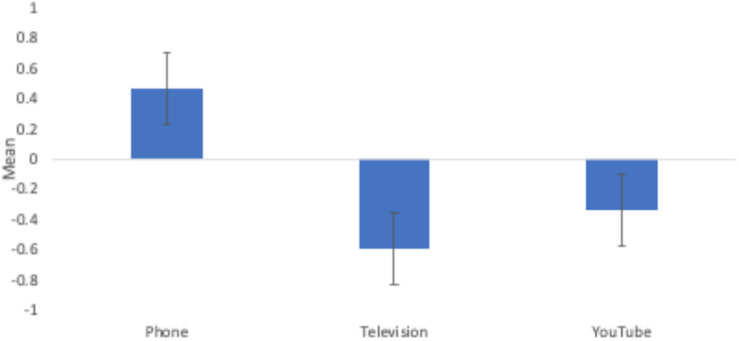
Children’s reality status judgments by medium.

To answer this question, we first checked for effects of medium order on children’s responses; children’s reality status judgments did not differ based on which of the three mediums they were asked about first (*p* > 0.165). We then conducted a one-way repeated measures ANOVA to compare the effect of age group, gender, media format, and the medium that children saw first on children’s reality status judgments. We were particularly interested in the interaction between media format and the medium that children saw first to determine whether the medium they were presented first differentially affected how the children responded to the three media formats. This interaction was non-significant (*p* = 0.313). Age group and gender were also not significant predictors or part of any significant interaction effects (*p* > 0.183) so they were dropped from the analysis.^1^ There was a main effect of media format, *F*(2,344) = 5.61, *p* = 0.004, η_p_^2^ = 0.032—showing that, as predicted, children were more likely to believe that the person in the phone video was real (*M* = 0.47) compared with the person from television (*M* = −0.59, *p* = 0.004). Children’s belief in the person from YouTube fell in between (*M* = −0.34), lower than the phone video (*p* = 0.044) and not significantly different from television (*p* = 1.00; see Figure 2)^2^. The age by condition interaction was not significant, indicating that our hypothesis was not supported: younger children seem to understand the differences between the media formats similarly to older children, with no apparent developmental change.

We further examined whether the mean score for each medium was significantly different from 0. Phone was significantly above 0, *t*(114) = 2.00, *p* = 0.024, and television was significantly below 0, *t*(116) = 2.51, *p* = 0.006, but YouTube did not significantly differ from 0, *t*(115) = 1.47, *p* = 0.071, suggesting that whereas children are likely to believe phone videos are real and television videos are not, there is less certainty about the status of YouTube videos.

#### How Do Children Justify Their Reality Status Judgments?

**FIGURE 3:**
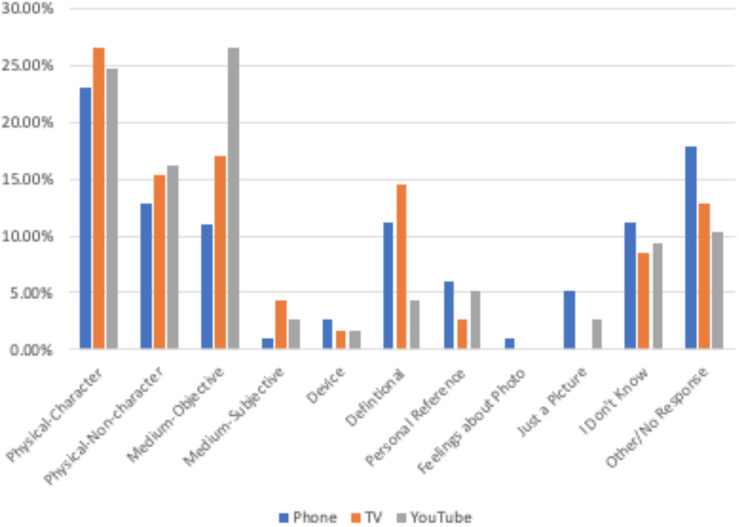
Percentage of children providing each justification by medium.

Figure 3 depicts children’s justifications for their reality status judgments by medium. For phone and television, children’s most popular justification was based on the physical characteristics of the person in the video (physical-person), such as, “He can’t be that tall.” (23.1% for phone and 26.5% for television). Although physical-person justifications were also prevalent for YouTube (24.8%), the most frequent justification was supplying fact or description of the medium (medium-objective) as a justification (26.5%), such as, “YouTubers are actually in real life, and they report on real things that really happen.”

Next, we conducted a series of one-way ANOVAs to examine the effect of age group and medium on each justification category. Belief score was dropped from these models, because there were no significant main effects of or interactions involving children’s reality status judgments on their justifications, *p* > 0.06, suggesting that children’s justifications did not differ based on whether they believed the person was real or not real. For medium-objective, there were effects of both medium, *F*(2,350) = 3.16, *p* = 0.044, η_p_^2^ = 0.018, and age group, (2,350) = 8.28, *p* < 0.001, η_p_^2^ = 0.046. Children gave this type of justification more often for YouTube (26.5%) than phone (11%, *p* = 0.040) but not significantly more than television (17.1%, *p* = 0.387). Additionally, both 5- and 6-year olds (18.9%, *p* = 0.007) and 7- and 8-year olds (26.1%), *p* < 0.001, gave this type of justification more than 3- and 4-year olds (1.7%). The age group by medium interaction was not significant, suggesting that the differences by medium were consistent across the age range.

**FIGURE 4:**
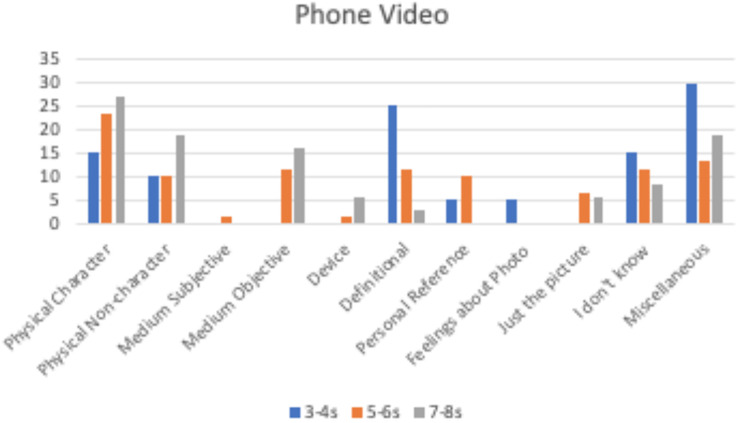
Percentage of children by age group providing each justification for the phone video.

**FIGURE 5:**
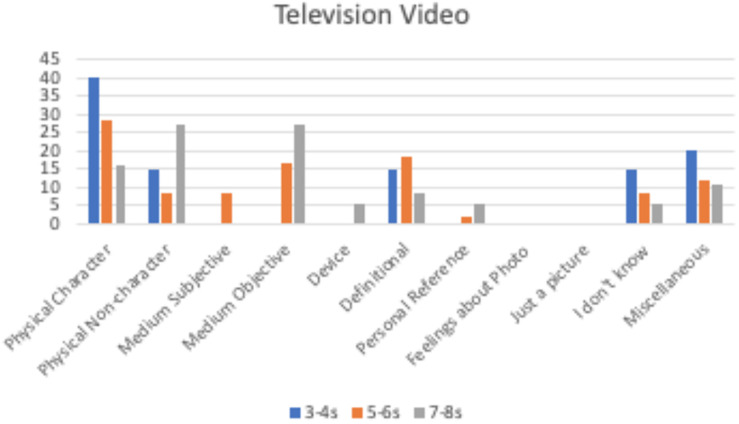
Percentage of children by age group providing each justification for the television video.

**FIGURE 6:**
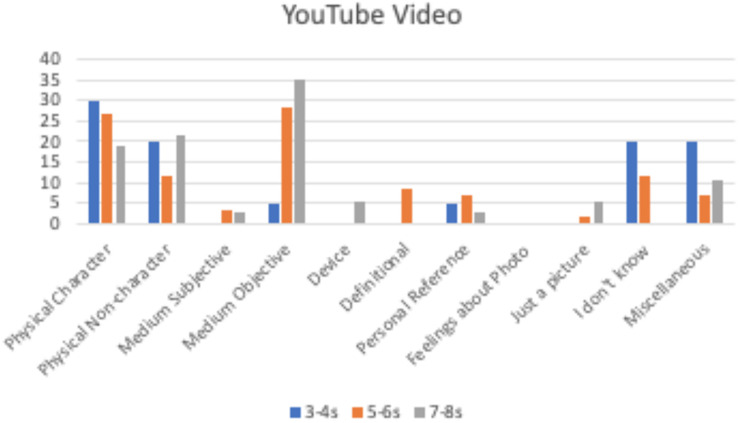
Percentage of children by age group providing each justification for the YouTube video.

Children were more likely to provide definitional justifications [directly addressing the definition of the question, such as, “I think he is real, because he looks, *F*(2,350) = 3.78, *p* = 0.024, η_p_^2^ = 0.022]. There was also a significant effect of age group for physical-non-person—a comment about something else in the picture or about the physical picture itself, such as, “The bird on the tree is small,” *F*(2,350) = 4.29, *p* = 0.014, η_p_^2^ = 0.024, with 7- and 8-year olds (22.5%) giving this type of justification more often than 5- and 6-year olds (10%, *p* = 0.011) with 3- and 4-year olds in the middle (15%), regardless of medium. There was a significant age group × medium interaction for feelings about the video, *F*(2,350) = 2.49, *p* = 0.043, η_p_^2^ = 0.028, with only 3- and 4-year olds providing this justification, and only for phone (5%). Additionally, the youngest children justified their responses with, “I don’t know,” (16.7%) more than 7- and 8-year olds (4.5%), *p* = 0.032, *F*(2,350) = 3.45, *p* = 0.033, η_p_^2^ = 0.020. Similarly, 3- and 4-year olds gave no response or responses outside the coding scheme more frequently (23.3%) than 5- and 6-year olds (10.6%), *p* = 0.039, *F*(2,350) = 3.12, *p* = 0.045, η_p_^2^ = 0.018. There were no significant differences based on age group, medium, or the interaction between the two for the other justifications, *p* > 0.06. Figures 4–6 depict the use of justifications by age group for each medium separately.

### Beliefs About Educational Value

#### How Does Media Format Affect Children’s Beliefs About the Educational Value of Videos?

Again, a one-way repeated measures ANOVA was conducted to compare the effect of media format on children’s beliefs about the educational value of videos, while also investigating the effects of age group. The outcome was an educational value score, ranging from −1 to 1 representing whether the child chose each medium first, second, or third. There was a significant main effect of medium, *F*(2,340) = 5.82, *p* = 0.003, η_p_^2^ = 0.034, with children regardless of age perceiving higher educational value for YouTube (*M* = 0.24) than phone (*M* = −0.09, *p* = 0.014) and television (*M* = −0.11, *p* = 0.008). There was no significant effect of age group (*p* = 0.981) or interaction between age group and medium (*p* = 0.118).

##### How do children justify their beliefs about a medium’s educational value?

Across mediums, children most frequently justified their educational value choice by supplying facts or descriptions of the medium (medium-objective; phone: 31%, television: 53.1%, and YouTube: 33.3%). Next, we conducted a series of one-way ANOVAs to examine the effect of age group and first medium chosen for educational value on each justification category. There was a significant interaction between age group and first medium chosen for the physical-non-person justification, *F*(2,116) = 3.13, *p* = 0.011, η_p_^2^ = 0.129, with younger children providing this type of justification more than older children, except for phone—where 5- and 6-year olds (80%) gave this type of justification more than both younger and older children (3–4 s: 20%; 5–6 s: 22.2%). For medium-objective, there was a significant main effect of age group, *F*(2,116) = 14.58, *p* < 0.001, η_p_^2^ = 0.216, which was driven by 7- and 8-year olds (67.6%) giving this justification more often than 3- and 4-year-olds (0%, *p* < 0.001) and 5- and 6-year olds (31.7%, *p* = 0.007). The youngest children (25%) gave no answer or a justification that did not fit the coding scheme more frequently than 5- and 6-year olds (5%, *p* = 0.006) and 7- and 8-year olds (8.1%, *p* = 0.004), *F*(2,116) = 5.09, *p* = 0.008, η_p_^2^ = 0.088. There were no significant individual predictors or interactions for the other justification categories (*p* > 0.06).

Similarly, across all three medium types, children were most likely to justify their third choice (that a video was not as good to learn from) based on providing facts or descriptions of the medium (medium-objective), phone = 21.1%; television = 29.5%, YouTube = 51.6%. We then conducted a series of one-way ANOVAs to examine the effect of age group and last choice medium preference on each justification category. There was a significant effect of age group for medium-objective, with 7- and 8-year olds (56.8%) selecting this justification more often than both 3- and 4-year olds (10%, *p* = 0.004) and 5- and 6-year olds (26.7%, *p* = 0.047). No significant individual predictors or interactions emerged for any of the other justification categories (*p* > 0.06).

#### How Do Children’s Beliefs About Educational Value Relate to Reality Status Judgments?

We conducted a regression model predicting children’s beliefs about educational value from age group, medium, belief score, and their interactions. There were no significant predictors or interactions (*p* > 0.107).

### Preference

#### How Does Media Format Affect Children’s Preferences for Videos?

To answer this question, a one-way repeated measures ANOVA was conducted to compare the effect of age group and media format on children’s preferences. Age group was not a significant predictor nor was it part of a significant interaction with medium (*p* > 0.10) so it was dropped from the analysis. There was a main effect for media format, *F*(2,347) = 8.41, *p* < 0.001, η_p_^2^ = 0.047, showing that children were more likely to prefer to watch YouTube (*M* = 0.22, *p* = 0.001) and television (*M* = 0.03, *p* = 0.049) than phone video (*M* = −0.25). There were no significant differences in preference between television and YouTube (*p* = 0.292).

##### How do children justify their medium preferences?

For first-choice preference, children who chose the phone video justified their selection most frequently by giving a fact or some element of description about the medium, such as, “Sometimes you can find all sorts of stuff on the phone, some of it is true and you can learn a lot from that stuff” (medium-objective; 23.5%), whereas children who preferred the television or YouTube video most frequently justified their choice using an opinion about the medium (medium-subjective; television: 27.5%; YouTube: 32.2%).

We then conducted a series of one-way ANOVAs to examine the effect of age group and first-choice medium preference on each justification category. There were no main effects or interactions with medium (*p* > 0.06), but there were some age effects. Regarding the medium-objective justification, there was a significant main effect of age group, *F*(2,115) = 5.46, *p* = 0.005, η_p_^2^ = 0.093, which was driven by 7- and 8-year olds (37.8%) giving this justification more often than 3- and 4-year olds (0%, *p* = 0.021) with 5- and 6-year olds falling in between (18.3%). There was also a significant main effect of age group for the Educational Information justification—or comments based on the educational quality of the medium, such as, “I prefer YouTube, because I can learn from it,” *F*(2,115) = 4.03, *p* = 0.021, η_p_^2^ = 0.070. Here, 7- and 8-year olds (13.5%) were more likely to use this justification than 5- and 6-year olds (1.7%, *p* = 0.035) and 3- and 4-year olds (0%, *p* = 0.050). There were no significant individual predictors or interactions for the other justification categories (*p* > 0.06).

Similarly, across all three medium types, children most frequently justified their decision that a video was their least favorite by providing facts or descriptions of that medium (medium-objective; phone = 17.6%; television = 15.4%, YouTube = 30.8%). We conducted a series of one-way ANOVAs to examine the effect of age group and last choice medium preference on each justification category. For both definitional, such as, “He is real, because he looks real,” *F*(2,115) = 2.99, *p* = 0.022, η_p_^2^ = 0.101, and just a photo—or comments about how it is just a photograph and not an actual video—justifications, *F*(2,115) = 2.99, *p* = 0.022, η_p_^2^ = 0.101, there were significant age group by medium interactions. Only 3- and 4-year olds used these two justifications (and only for television, 16.7% for definitional, 16.7% for just a photo) for these questions—no other age groups used them for any medium. No significant individual predictors or interactions emerged for any of the other justification categories, *p* > 0.06.

#### How Does Children’s Preference for a Particular Format Relate to Reality Status Judgments?

We conducted a regression model predicting children’s preference from their reality status judgments, age group, medium, and their interaction. Children’s belief scores significantly predicted their preferences, *b* = 0.491, *t*(6) = 2.23, *p* = 0.026, with children having a greater preference for videos that they believed to be more real (*r* = 0.111, *p* = 0.002). There were no significant predictors or interactions, *p* > 0.152.

#### How Does Children’s Preference Relate to Their Belief About Educational Value?

Finally, we conducted a regression model predicting children’s beliefs about educational value from age group, preference, and their interactions. Preference significantly predicted children’s beliefs about a medium’s education value, *b* = 0.46, *t*(6) = 2.54, *p* = 0.011, meaning that children believed a medium had more educational value when they also had a greater preference for it (*r* = 0.507, *p* < 0.001). No other predictors or interactions were significant, *p* > 0.500.

## Discussion

The goal of this study was to examine how different media formats affect children’s reality status judgments, preferences, and beliefs about videos’ educational value. The COVID-19 pandemic has accelerated children’s use of YouTube for both entertainment and educational purposes. As a result, research investigating how children conceptualize the people they view on YouTube is even more imperative than ever. Are these people real—like caregivers and friends? Or are they not real—like people on television?

### Reality Status Judgments

As we predicted, children recognized that the phone video was more likely to be real than television, suggesting that they understood and followed our procedure and questions. YouTube fell in between phone and television, confirming the idea that YouTube may be a murkier area for children to understand reality status, perhaps given the diverse content on the platform. That it was not rated as more real than television suggests that children may default to believing screen content is not real (Woolley and Ghossainy, 2013) and may not fully appreciate YouTube’s intermediate status.

Wright et al. (1994) noted that children use form and context clues to help them determine the reality status of television. In this study, we coded children’s reality status justifications using 11 different categories that focused on similar areas—as well as others—to determine why children made their judgments. Interestingly, children tended to justify their reality status judgments for YouTube by referring to objective characteristics of the medium more than for either television or phone. This finding, along with YouTube’s intermediate status in children’s reality status scores, suggests that children may find YouTube a more complex medium and thus are really thinking about features of the medium itself to make their judgments. Judgments for television and phone may seem more obvious to children and thus make it more difficult for them to verbalize their justifications. Furthermore, neither children’s reality status judgments nor their use of this type of justification changed with age suggesting that children’s basic understanding of the reality status of YouTube does not develop significantly across this large age range—even the youngest children in our sample (3- and 4-year olds) demonstrated a familiarity with the platform and made similar judgments about its reality status as 7- and 8-year olds. This highlights that even young preschoolers are familiar with YouTube and are able to make similar judgments about it as children more than twice their age.

### Beliefs About Educational Value

Regardless of age, children perceived greater educational value in YouTube as compared with both phone and television. This is striking, given the plethora of educational content available on television and the diverse content present on YouTube. It is not clear why children see YouTube as a better learning source. Perhaps differences in the content children view on YouTube accounts for the finding, such as how-to-videos, which are watched by 38% of 0–8-year olds (Common Sense Media, 2017) and can help children learn all kinds of things—from rollerblading to math problems. It might be that we see children’s beliefs about YouTube’s educational value increase even further due to the COVID-19 pandemic, since many educators have been posting videos to support children’s at-home learning. Future research should obtain more detailed information about children’s viewing habits to assess this possibility.

It was also striking that there were no age-related differences regarding the educational value of the various media. Previous television research suggests that older children are better at recognizing the nuances of television programs, with some being real and some not (Wright et al., 1994), which may result in a clearer understanding of learning potential. Yet, we found that children made similar choices regardless of age. It may be that YouTube—in its novelty—cleaves less closely to traditional distinctions in reality versus non-reality and educational value, which leads to weaker societal beliefs about its purpose. Though, when considering children’s justifications of educational value, the oldest children overwhelmingly chose medium-objective reasons to justify their beliefs. This suggests that they may have a more advanced grasp on the nature of the various media types, which is in line with expected age-related differences.

Additionally, children’s reality status judgments did not predict their beliefs about a medium’s educational value. This lack of relation between children’s assessment of reality status with their judgments about educational value is aligned with Hawkins (1977) finding that younger children believed that television characters were more real than their older peers, but they did not endorse statements about the educational utility of television, such as, “Watching police officers on television helps me understand the police I might meet.” However, this finding is somewhat surprising given research by Mares and Sivakumar (2014) and Bonus and Mares (2019) showing that children are less likely to learn from television when they judge that a show’s content is not real. It may be the case that children’s judgments of educational value and their actually ability to learn from media content do not always go hand in hand. Future research should explore both learning and perceptions of educational value together, as our results did show children’s perceptions of educational value were linked to their preference and interest in watching the video.

### Preference

Perhaps not surprisingly, regardless of age, children preferred YouTube and television over phone videos, suggesting that children make assumptions about the quality or interest level of videos based on platform. Notably, children preferred to watch videos that they believed were real. This finding appears to align with previous research about children’s distinctions between reality and fantasy. As early as the preschool years, children are able to identify the difference between real and fantastical people and characters (Skolnick and Bloom, 2006). They are also able to attribute necessary human functions, such as needing to eat, to real people and not to fantastical ones (Sharon and Woolley, 2004). In general, children possess some amount of disbelief about fantastical contexts, which might result in not preferring to watch them (see also Lillard and Taggart, 2019).

Interestingly, for children’s last choice preference, only 3- and 4-year olds gave just a picture justifications—and only for television. Comments about the picture itself, such as, “It’s just a picture,” may represent children’s inability to look beyond the image presented to them to see the medium that is being represented. This is in line with research by Flavell et al. (1990), which suggests that 3-year olds view images from television as real objects, while older children are able to understand these images are representations of objects.

Importantly, preference positively predicted educational value for all media, suggesting that children may be more interested in videos that offer to-be-learned content on these platforms. This is notable given the demonstrated value of educational media content in promoting children’s skills (e.g., Mares and Pan, 2013; Hurwitz, 2018). Although entertainment media is popular among children, our findings suggest that when content is matched, children prefer videos that they can learn from. Research with storybooks also suggests that children might prefer to interact with media that help them learn new information. When preschool children were read two matched books—one with detailed causal information such as why animals behave and look in certain ways—and another that simply described animals and their behaviors, Shavlik et al. (2020) found that children preferred the causally rich storybook, perhaps because they found it more engaging.

### Limitations

One limitation of the current study is the lack of racial/ethnic diversity in the sample (61.5% white children). There was greater socioeconomic diversity present within the sample, but ideally, the sample would contain a larger percentage of children from underrepresented populations. This may be important because African–American and Latinx children spend more time using mobile media compared with white children (Rideout, 2017) and thus may have higher exposure to YouTube content, which may influence perceptions.

Another limitation might have been the image used as the study stimuli. The image was very deliberately created—a male-appearing person was chosen because approximately 62% of YouTube users/creators are male (Drazovic, 2019), and a bird was selected as the animal in the image since children from rural, suburban, and urban environments all encounter birds in the course of their everyday lives. This procedure was extensively pilot tested to ensure that children were able to attend to the format of the video instead of just focusing on the image itself, and only one child commented that the images for all three media types were the same. Additionally, 82% of children made comments based on medium, which strongly suggests that they were able to attend to the different platforms presented. However, because we only test one type of image, generalizability may be limited and results may not extend to other types of videos.

Another limitation is that our study asked about children’s perceptions of reality status and educational potential, rather than assessing their learning from different mediums. Yet, research shows that preschool-age children are less likely to learn from television when they judge that content is not real (Mares and Sivakumar, 2014), so exploring the relation between children’s reality status judgments and their beliefs about educational value may be valuable for furthering our understanding of this phenomenon. Future studies should investigate how children are actually able to learn from YouTube videos, as opposed to only measuring how much they believe they can learn from them, and explore links to their reality status judgments.

It is also likely that children do not conceptualize the differences between media types in the same way that adults do. They may be motivated primarily to find the content that they enjoy watching and not care about the platform on which they can view that content. That being said, results did show that children’s preference did positively predict educational value for all media, suggesting that children may in fact be most interested in videos that offer educational content—no matter what the platform.

Furthermore, we used a laboratory-based procedure and researcher-created image to maximize experimental control, but we may have missed important elements of children’s YouTube viewing experience by controlling content across platforms.

## Conclusion

As a result of the COVID-19 pandemic, many early childhood and K–12 schools moved to online instruction with only a few days’ notice. Video conference class meetings and YouTube videos of lessons and storybook readings supplanted classroom instruction and radically changed the educational landscape across the globe (Li and Lalani, 2020). However, research is lacking on how children conceptualize people that they view on YouTube. This study aimed to describe how children aged 3–8 make judgments about media’s reality status, determine their preferences, and reason about videos’ educational value. Results suggest that YouTube does occupy a unique space in children’s media landscape. Children are more likely to see YouTube content as educational, which might help them learn more from educational content on the platform.

Media literacy curricula will do well to include information specific to YouTube and other online video platforms, given their popularity among children in recent years. In the context of the current pandemic and with the possibility of future spikes, online learning modalities are likely to be a part of children’s educational experiences for months and years to come. Knowledge regarding the power of YouTube for education will help educators and caregivers make informed decisions for children’s success.

## Data Availability Statement

The datasets generated for this study can be found in online repositories. The names of the repository/repositories and accession number(s) can be found below: https://osf.io/n37z6/?view_only=87a1fc1c161548f9a289270a6b3c751c.

## Ethics Statement

The studies involving human participants were reviewed and approved by Pace University IRB and The Ohio State University IRB. Written informed consent to participate in this study was provided by the participants’ legal guardian/next of kin.

## Author Contributions

BH-D led the design the study, conducted data analysis, and led the writing of the manuscript. RD helped to design the study and consulted on analyses and helped to write the manuscript. KA conducted all of the justifications coding and helped to write the manuscript. MH and MPe conducted data collection and assisted in writing the manuscript. MPa conducted data collection and helped to design the coding scheme for the justifications. All authors contributed to the article and approved the submitted version.

## Conflict of Interest

The authors declare that the research was conducted in the absence of any commercial or financial relationships that could be construed as a potential conflict of interest.

## Footnotes

1 Gender and medium that children saw first were not a significant predictor for any of the other analyses presented here (*p* > 0.313), so they were dropped from all subsequent analyses and will not be discussed further.

2 All factors, factor interactions, and *post hoc* comparisons described in this manuscript were corrected for type I error using the Bonferroni correction.
